# The association between occupational secondhand smoke exposure and life satisfaction among adults in the European Union

**DOI:** 10.1186/s12971-017-0127-x

**Published:** 2017-03-23

**Authors:** Nikita B. Rajani, Iris T. Vlachantoni, Constantine I. Vardavas, Filippos T. Filippidis

**Affiliations:** 1 Department of Primary Care and Public Health, School of Public Health, Imperial College London, 310 Reynolds Building, St. Dunstan’s Road, London, W6 8RP United Kingdom; 2 National Heart and Lung Institute, Imperial College London, London, United Kingdom; 3 Clinic of Social and Family Medicine, School of Medicine, University of Crete, Heraklion, Greece; 4 European Network for Smoking and Tobacco Prevention (ENSP), Brussels, Belgium; 5 Center for Health Services Research, School of Medicine, National and Kapodistrian University of Athens, Athens, Greece

**Keywords:** Life satisfaction, Tobacco, Secondhand smoke, Occupational, Europe, Smoking, Passive smoking

## Abstract

**Background:**

Despite existing legislation, a large proportion of the European Union (EU) population is exposed to occupational secondhand smoke (SHS). The aim of this study was to explore associations between occupational exposure to SHS and self-reported life satisfaction.

**Methods:**

We analysed data collected through the Eurobarometer survey (wave 82.4) from *n* = 11,788 individuals working in indoor spaces. The sample was representative of the population of the 28 EU member states. We fitted a multilevel logistic regression model adjusting for smoking, age, gender, occupation, area of residence, education, difficulty paying bills, marital status and social class.

**Results:**

27.5% of those working indoors reported at least some occupational exposure to SHS. People exposed to occupational SHS were less likely to report that they were satisfied with the life they lead (adjusted Odds Ratio = 0.72, 95% Confidence Interval: 0.60-0.87). The effect of occupational exposure on life satisfaction did not differ by smoking status, with all interaction terms between smoking status and occupational exposure to SHS not statistically significant.

**Conclusion:**

Exposure to SHS at the workplace does not only have negative consequences on physical health, but it can also impact life satisfaction of smokers and non-smokers. Our findings highlight the need for stricter enforcement of smokefree environments at the workplace in the EU.

**Electronic supplementary material:**

The online version of this article (doi:10.1186/s12971-017-0127-x) contains supplementary material, which is available to authorized users.

## Introduction

Exposure to secondhand smoke (SHS) has been highlighted as a contributing factor in the pathogenesis of many diseases including lower respiratory infections, otitis media, asthma, lung cancer and ischaemic heart disease. SHS is also a significant cause of worldwide mortality and Disability-adjusted life years (DALYs) lost [1]. Exposure to SHS has declined as a potential risk factor due to legislative actions and changing patterns in population health and disease [1]. All members of the European Union (EU) have some kind of smoke-free legislation [2], but as many as three in ten non-smokers are still exposed to SHS in indoor areas in the EU [3]. Hence, despite the fact that the Framework Convention on Tobacco Control (FCTC) recognized SHS as a public health threat and called for effective measures to control it, reducing exposure to SHS remains a challenge [4]. Within the EU there is considerable discrepancy between and within EU states in how this commitment translates into policy development and law implementation, with almost one in four (24.4%) adults reporting occupational exposure to SHS [3]. Thus, the EU population is still exposed to SHS with potentially detrimental effects to their physical health and, as recent data suggest, their mental wellbeing [5].

The current research agenda and literature tends to examine the consequences of SHS exposure on physical health, stemming from the lack of data regarding the existing psychological impact. However, recent studies, primarily from Asian countries, point to a potentially significant association between occupational SHS exposure and depression or stress [6–9]. While life satisfaction is a concept distinct from mental health or wellbeing, it has been causally linked to major depression, anxiety disorder, and suicidality [10], as well as with mortality [11, 12]; therefore it may serve as an indicator of the impact that exposure to SHS may have on individuals. The aim of this study was to explore the association between occupational exposure to SHS and self-reported life satisfaction among people aged 18 years or older in the 28 EU member states in late 2014.

## Methods

### Data source

We analysed data from wave 82.4 of the Eurobarometer survey, which was conducted in November-December 2014 in all 28 EU member states [13]. A total of 27,801 individuals aged ≥15 years were interviewed at their homes. The sample was selected through multi-stage random sampling and was representative of the EU population aged 15 years or older in terms of age, gender and area of residence. Primary sampling units (PSU) were selected from each region within each member state, proportional to population size. A sample of starting addresses was randomly selected in each PSU, and households were systematically selected following a standard random route. Following the collection of the data, post-stratification and population size weighting were applied in each member state using Eurostat data on gender, age and area of residence. We only analysed data of individuals aged ≥18 years, considering that reported occupation among adolescents may be transient and potential exposure to SHS likely to vary within short periods of time.

### Measures

Occupational exposure to SHS was evaluated among Eurobarometer respondents who reported to be working at the time of the survey who were asked “How often are you exposed to tobacco smoke indoors at your workplace?”. Responses were grouped into exposed (“occasionally”; “less than 1 h a day”; “1 to 5 h a day”; “more than 5 h a day”) and not exposed (“never or almost never”).

Self-reported life satisfaction was assessed with the question “On the whole, are you very satisfied, fairly satisfied, not very satisfied or not at all satisfied with the life you lead?” and responses were grouped into “satisfied” (very or fairly satisfied) and “not satisfied” (not very or not at all satisfied).

Tobacco smoking was assessed with the question “Regarding smoking cigarettes, cigars or a pipe, which of the following applies to you?”. Those who responded that they had never smoked were classified as never smokers and those who said that they used to smoke but they had stopped were classified as ex-smokers. Respondents who said that they currently smoked were further asked how many cigarettes they smoke each day. Based on their response, current smokers were classified as light smokers (<10 cigarettes per day), moderate smokers (10–19 cigarettes per day) and heavy smokers (≥20 cigarettes per day).

The survey also collected data on the respondents’ age (18–24; 25–39; 40–54; or ≥55 years); gender (female; or male); area of residence (urban; or rural); difficulty to pay bills (never/almost never; or from time to time/most of the time); age at which they stopped full-time education (≤15; 16–19; or ≥20 years); marital status (married/single living with partner; unmarried; or divorced/separated/widowed); self-reported social class (working class; lower middle class; middle class; or upper middle/higher class), and occupation (manual workers; other non-manual workers; self-employed; and managers), parameters which were evaluated as potential covariates.

We also estimated the prevalence of smoking among adults at a country level, using the Eurobarometer dataset. We used the Tobacco Control Scale (TCS) to extract data on smoke-free policies at a country level and included the scores of the “smoke-free work- and other public places” subscale in the analysis. The highest score that can be achieved by a country in this subscale is 22 [14].

### Statistical analysis

We used a multilevel logistic regression model to assess the association between occupational exposure to SHS and self-reported life satisfaction. All socio-demographic variables described earlier, as well as smoking status, smoke-free TCS score and smoking prevalence at a country level were used as covariates in the model. Reference groups for all variables were defined as either the “lowest” category (ordinal variables) or the category with the highest number of individuals (nominal variables). We also included an interaction term between smoking status and exposure to SHS in the model –variable classification as described above- in order to explore potentially differential impact of occupation SHS exposure to life satisfaction by smoking status. A sensitivity analysis excluding all those <25 years of age was also conducted, in order to explore whether the associations differ among those with -assumed- more permanent occupations. Logistic regression results are presented as adjusted Odds Ratios (aOR) with 95% Confidence Interval (CI). Official weights provided in the publicly available dataset were used for the descriptive analyses in order to take the complex study design into account.

## Results

Sample characteristics are shown in Additional file 1: Table S1. A total of 27.5% of the 11,788 adults working indoors reported at least some occupational exposure to SHS. Occupational exposure to SHS was associated with a lower likelihood of reporting life satisfaction (aOR = 0.72, 95% CI: 0.60–0.87) (Table 1). Those of younger age were more likely to report life satisfaction than those of older age (aOR = 1.64, 95% CI: 1.20–2.24), as were those who reported themselves to be of upper social class (aOR = 3.49, 95% CI: 2.48–4.90) or higher educated (aOR = 1.54, 95% CI: 1.23–1.94). The interaction between the respondents’ smoking status and occupational exposure to SHS was evaluated but was not identified as statistically significant after adjusting for all covariates. Current smokers were less likely to report feeling satisfied with their lives compared to never smokers (aOR = 0.60, 95% CI: 0.47–0.76 for heavy smokers). Results from the sensitivity analysis (among those aged ≥25 years) were similar to the main analysis.

**Table 1:** Factors associated with life satisfaction amongst employed individuals in 28 European Union Member States (*N* = 11,788)

	Life satisfaction	
Outcome: life satisfaction	Unadjusted OR (95% CI)	Adjusted OR (95% CI)
Occupational SHS exposure		
No (Ref.)	1.00	1.00
Yes	0.70 (0.63 – 0.79)	0.72 (0.60 – 0.87)
Smoking status/occupational SHS exposure		
Never smoker * exposed to SHS(Ref.)	1.00	1.00
Ex-smoker * exposed to SHS	0.91 (0.67 – 1.26)	0.99 (0.69 – 1.41)
Light smoker * exposed to SHS	1.39 (0.86 – 2.25)	1.57 (0.92 – 2.68)
Moderate smoker * exposed to SHS	1.50 (1.09 – 2.06)	1.30 (0.92 – 1.85)
Heavy smoker * exposed to SHS	1.26 (0.92 – 1.72)	1.21 (0.86 – 1.70)
Smoking status		
Never smoker (Ref.)	1.00	1.00
Ex-smoker	1.04 (0.90 – 1.21)	1.09 (0.89 – 1.34)
Light smoker (<10 cigarettes per day)	0.76 (0.61 – 0.94)	0.71 (0.53 – 0.96)
Moderate smoker (10–20 cigarettes per day)	0.66 (0.57 – 0.77)	0.76 (0.62 – 0.93)
Heavy smoker (≥20 cigarettes per day)	0.46 (0.39 – 0.54)	0.60 (0.47 – 0.76)
Age (years)		
≥ 55 (Ref.)	1.00	1.00
40-54	1.03 (0.90 – 1.18)	1.03 (0.88 – 1.20)
25-39	1.53 (1.21 – 1.77)	1.56 (1.31 – 1.86)
18-24	1.39 (1.07 – 1.80)	1.64 (1.20 – 2.24)
Gender		
Female (Ref.)	1.00	1.00
Male	1.04 (0.93 – 1.15)	1.05 (0.93 – 1.18)
Area of residence		
Rural (Ref.)	1.00	1.00
Urban	1.01 (0.90 – 1.13)	0.91 (0.79 – 1.03)
Difficulty in paying bills		
Never/Almost never (Ref.)	1.00	1.00
Time to time/Most of the time	0.22 (0.19 – 0.24)	0.28 (0.25 – 0.32)
Age when full-time education was stopped (years)		
≤ 15 (Ref.)	1.00	1.00
16-19	1.92 (1.59 – 2.31)	1.35 (1.10 – 1.66)
20+	3.43 (2.82 – 4.17)	1.54 (1.23 – 1.94)
Marital status		
Married/Cohabitation (Ref.)	1.00	1.00
Unmarried	0.60 (0.52 – 0.70)	0.57 (0.48 – 0.67)
Divorced/Separated/Widowed	0.33 (0.29 – 0.39)	0.45 (0.38 – 0.53)
Social class		
Working class (Ref.)	1.00	1.00
Lower Middle class	1.27 (1.10 – 1.47)	1.10 (0.94 – 1.29)
Middle class	3.57 (3.14 – 4.06)	2.38 (2.05 – 2.76)
Upper middle/higher class	7.09 (5.20 – 9.67)	3.49 (2.48 – 4.90)
Occupation		
Manual workers (Ref.)	1.00	1.00
Other non-manual workers	1.64 (1.44 – 1.86)	1.16 (0.99 – 1.35)
Self-employed	1.58 (1.36 – 1.85)	1.17 (0.97 – 1.40)
Managers	2.43 (2.08 – 2.84)	1.14 (0.94 – 1.39)
Tobacco Control Scale Smoke-free score (per 1-point increase)	1.09 (1.00 – 1.18)	1.06 (0.99 – 1.13)
Smoking prevalence (per a 1% increase)	0.89 (0.85 – 0.94)	0.93 (0.89 – 0.98)

## Discussion

The main finding of our analysis indicates that occupational exposure to SHS was associated with lower life satisfaction amongst employed individuals in the 28 EU member states. Since life satisfaction is robustly associated with mental health problems [10], our finding is in agreement with prior studies that have examined the impact of SHS on mental health [7–9]. One possible biological explanation for this finding could be that SHS exposure is linked with low-grade inflammation which is strongly associated with mental health problems, particularly mood disorders like depression [15]. Another plausible reason could be related to the known effect of nicotine on psychophysiological pathways relevant to mental health such as the dopaminergic system or adrenocortical function [5]. However, this association that was identified might have a social element as well. It was initially hypothesised that those who were mostly exposed to occupational SHS were of lower socio-economic level and/or involved in manual labour employment [16], in which case their socio-economic circumstances could partly explain their lower life satisfaction in comparison to their peers. However, in the analysis we did adjust for factors such as occupation and social class, and the findings indicated that exposure to occupational SHS was unrelated to occupation.

Aside from the aforementioned implications on the mental health of individuals exposed to occupational SHS, this finding also has implications on work productivity. Since life satisfaction is often used as subjective indicator for work productivity, lower satisfaction implies decreased work productivity, which in turn has negative consequences on output [17].

Interestingly, we found that occupational exposure to SHS had the same impact on life satisfaction for smokers and non-smokers, which suggests that smokers are not more tolerant to SHS than non-smokers. This may be a result of slightly different pathways in smokers and non-smokers; for smokers, it might mean that they are also allowed to smoke at work and therefore are exposing themselves to greater risks, while for non-smokers it is more likely a direct result of being exposed to SHS, which may not only cause them discomfort, but also worsen their physical health and thus negatively affect their life satisfaction. Regardless of the pathway and possible explanations though, it is important to highlight that exposure to SHS is equally harmful, at least in the domain we assessed, to all people, regardless of whether they smoke or not [18].

One of the main strengths of our analysis is that the sample is large and representative of the EU population. Since there have not been studies examining the relationships tested within the EU, our findings add to the existing body of literature on the impact of SHS in Europe and emphasize the need to enforce effective legislation to protect the mental health of individuals. Despite these strengths, exposure to SHS might not be accurately measured as existing literature indicates that self-reporting of SHS exposure can potentially be an imprecise measure of biologically confirmed SHS exposure [19]. Furthermore, even though life satisfaction acknowledges a more positive aspect of mental health rather than the mere absence of disease, it is only measured using a single item in the Eurobarometer questionnaire. This may not be as robust of a measurement compared to other life satisfaction assessment tools such as the Satisfaction with Life (SWL) scale or the Life Satisfaction Questionnaire (LISAT-9), but it is in line with measures used for cross-country comparisons, such as the one adopted by the Organisation for Economic Co-operation & Development (OECD) [20]. Finally, while we cannot rule out unmeasured confounding, we were able to adjust for several individual level factors that could potentially influence life satisfaction which strengthens the external validity of our findings and we took into account the prevalence of smoking and the level of smoke-free policies at a country level.

In conclusion, we identified an interesting association between occupational exposure to SHS and life satisfaction across the EU population, a factor primarily noted to date within Asian populations in the literature. Further research is needed to confirm this finding and also identify the social or biological factors that mediate the association.

## Additional file

Additional file 1: Table S1. Sample characteristics (*N* = 11,788). (DOCX 16 kb)

## Abbreviations

aOR: Adjusted Odds Ratio
CI: Confidence Interval
DALYs: Disability-adjusted life years
EU: European Union
FCTC: Framework Convention on Tobacco Control
LISAT-9: Life Satisfaction Questionnaire
SHS: Secondhand smoke
SWL: Satisfaction with Life

## References

[1] Collaborators GBDRF, Forouzanfar MH, Alexander L, Anderson HR, Bachman VF (2015). Global, regional, and national comparative risk assessment of 79 behavioural, environmental and occupational, and metabolic risks or clusters of risks in 188 countries, 1990–2013: a systematic analysis for the Global Burden of Disease Study 2013. Lancet.

[2] World Health Organization (2015). WHO Report on the Global tobacco Epidemic 2015: Raising taxes on tobacco.

[3] Filippidis FT, Agaku IT, Girvalaki C, Jimenez-Ruiz C, Ward B (2016). Relationship of secondhand smoke exposure with sociodemographic factors and smoke-free legislation in the European Union. Eur J Public Health.

[4] World Health Organisation (2013). Parties to the WHO Framework Convention on Tobacco Control.

[5] Hamer M, Stamatakis E, Batty GD (2010). Objectively assessed secondhand smoke exposure and mental health in adults: cross-sectional and prospective evidence from the Scottish Health Survey. Arch Gen Psychiatry.

[6] Nakata A, Takahashi M, Ikeda T, Hojou M, Nigam JA (2008). Active and passive smoking and depression among Japanese workers. Prev Med.

[7] Jung SJ, Shin A, Kang D (2015). Active smoking and exposure to secondhand smoke and their relationship to depressive symptoms in the Korea national health and nutrition examination survey (KNHANES). BMC Public Health.

[8] Kim SJ, Han KT, Lee SY, Chun SY, Park EC (2015). Is secondhand smoke associated with stress in smokers and non-smokers?. BMC Public Health.

[9] Ye X, Li L, Gao Y, Zhou S, Yang Y (2015). Dose–response relations between second-hand smoke exposure and depressive symptoms among middle-aged women. Psychiatry Res.

[10] Fergusson DM, McLeod GF, Horwood LJ, Swain NR, Chapple S (2015). Life satisfaction and mental health problems (18 to 35 years). Psychol Med.

[11] Lacruz ME, Emeny RT, Baumert J, Ladwig KH (2011). Prospective association between self-reported life satisfaction and mortality: Results from the MONICA/KORA Augsburg S3 survey cohort study. BMC Public Health.

[12] Koivumaa-Honkanen H, Honkanen R, Viinamaki H, Heikkila K, Kaprio J (2000). Self-reported life satisfaction and 20-year mortality in healthy Finnish adults. Am J Epidemiol.

[13] European Commission (2014). Eurobarometer 82.4, November-December 2014. GESIS Data Archive: ZA5933, dataset version 5.0.0 (2014).

[14] Joossens LR M (2013). The Tobacco Control Scale 2013 in Europe.

[15] Jefferis BJ, Lowe GD, Welsh P, Rumley A, Lawlor DA (2010). Secondhand smoke (SHS) exposure is associated with circulating markers of inflammation and endothelial function in adult men and women. Atherosclerosis.

[16] Commission on Social Determinants of Health (CSDH) (2008). Closing the Gap in a Generation: Health Equity Through Action on the Social Determinants of Health.

[17] Halpern MT, Shikiar R, Rentz AM, Khan ZM (2001). Impact of smoking status on workplace absenteeism and productivity. Tob Control.

[18] U.S. Department of Health and Human Services. The Health Consequences of Involuntary Exposure to Tobacco Smoke: A Report of the Surgeon General. Atlanta GA: U.S. Department of Health and Human Services, Centers for Disease Control and Prevention, Coordinating Center for Health Promotion, National Center for Chronic Disease Prevention and Health Promotion, Office on Smoking and Health; 2006.

[19] Arheart KL, Lee DJ, Fleming LE, LeBlanc WG, Dietz NA (2008). Accuracy of self-reported smoking and secondhand smoke exposure in the US workforce: the National Health and Nutrition Examination Surveys. J Occup Environ Med.

[20] Organisation for Economic Co-operation & Development, OECD Better Life Index. http://www.oecdbetterlifeindex.org. Accessed 25 Aug 2016.

