# Supplementary material for: The association between occupational secondhand smoke exposure and life satisfaction among adults in the European Union
Source: Tob Induc Dis. 2017 Mar 23;15:19. doi: 10.1186/s12971-017-0127-x (PMC5364630; doi:10.1186/s12971-017-0127-x)
Supplement: Additional file 1: Table S1. — Sample characteristics (N = 11,788). (DOCX 16 kb) [file 12971_2017_127_MOESM1_ESM.docx]

Supplementary Table 1. Sample characteristics (N=11,788)

|  | n (weighted %) |
| --- | --- |
| Occupational SHS exposure |  |
| No | 8,560 (72.5) |
| Yes | 3,228 (27.5) |
| Smoking status |  |
| Never smoker | 6,101 (51.4) |
| Ex-smoker | 2,379 (18.9) |
| Light smoker (<10 cigarettes per day) | 687 (6.4) |
| Moderate smoker (10-20 cigarettes per day) | 1,403 (13.5) |
| Heavy smoker (≥20 cigarettes per day) | 1,218 (9.7) |
| Age (years)  ≥ 55  40-54  25-39  18-24 |  |
|  | 2,303 (15.6) |
|  | 4,933 (40.3) |
|  | 3,970 (36.7) |
|  | 582 (7.4) |
| Gender  Female |  |
|  | 6,044 (44.8) |
| Male | 5,744 (55.2) |
| Area of residence  Rural |  |
|  | 3,413 (28.8) |
| Urban | 8,371 (71.2) |
| Difficulty in paying bills  Never/Almost never |  |
|  | 7,589 (67.3) |
| Time to time/Most of the time | 4,083 (32.7) |
| Age when full-time education was stopped (years)  ≤15  16-19 |  |
|  | 807 (7.9) |
|  | 5,488 (50.1) |
| 20+ | 5,280 (42.0) |
| Marital status  Married/Cohabitation  Unmarried |  |
|  | 8,622 (71.6) |
|  | 1,813 (18.5) |
| Divorced/Separated/Widowed | 1,287 (9.9) |
| Social class  Working class  Lower Middle class  Middle class |  |
|  | 3,071 (27.7) |
|  | 1,828 (20.2) |
|  | 5,511 (43.5) |
| Upper middle/higher class | 1,096 (8.5) |
| Occupation |  |
| Manual workers  Other non-manual workers  Self-employed  Managers | 4,721 (40.8)  2,974 (24.2)  1,644 (15.0)  2,449 (20.0) |
